# The Outcomes of Total Hip Replacement in Patients with Parkinson's Disease: Comparison of the Elective and Hip Fracture Groups

**DOI:** 10.1155/2017/1597463

**Published:** 2017-09-27

**Authors:** Pavel Šponer, Tomáš Kučera, Michal Grinac, Aleš Bezrouk, Daniel Waciakowski

**Affiliations:** ^1^Department of Orthopaedic Surgery, Charles University in Prague, Faculty of Medicine in Hradec Králové, Šimkova 870, 500 38 Hradec Králové, Czech Republic; ^2^Department of Orthopaedic Surgery, University Hospital Hradec Králové, Sokolská 581, 500 05 Hradec Králové, Czech Republic; ^3^Department of Medical Biophysics, Charles University in Prague, Faculty of Medicine in Hradec Králové, Šimkova 870, 500 38 Hradec Králové, Czech Republic; ^4^Department of Orthopaedics and Traumatology, Kreiskrankenhaus Greiz GmbH, Wichmannstraße 12, 07973 Greiz, Germany

## Abstract

**Introduction:**

The aim of the study was to compare the clinical outcomes following elective and traumatic total hip arthroplasty in Parkinson's disease patients.

**Materials and Methods:**

Ten patients with osteoarthritis comprise the elective group (mean age at operation 74 years; mean follow-up 82 months). Thirteen patients with femoral fracture comprise the hip fracture group (mean age 76 years; mean follow-up 54 months). All patients were followed up at 6 and 36 months postoperatively and at the time of the latest follow-up.

**Results:**

Despite the significant improvement in Merle d'Aubigné-Postel and pain scores, disability related to Parkinson's disease increased during the follow-up. Whereas more than 1/3 of hip fracture patients and all elective patients walked independently at 36 months after total hip arthroplasty, 43% of living patients from both groups were able to walk independently at the time of the latest follow-up. The medical complications were seen mainly in patients with hip fracture.

**Conclusions:**

Excellent pain relief with preserved walking ability without support of another person and acceptable complication profile was observed in Parkinson's disease patients at 36 months after elective total hip arthroplasty. This procedure may be indicated in Parkinson's disease patients after careful and individualized planning.

## 1. Introduction

The prevalence of Parkinson's disease and the incidence of hip fractures mirror an ageing population living longer [1, 2]. Parkinson's disease patients with hip fractures stay at higher risk of mortality and surgical and medical complications [3]. Short- and long-term results in patients with Parkinson's disease following hip fracture are generally described to be worse than in patients without this disease [4]. The advancement in pharmacotherapy and surgical treatment has improved the life spans in patients with Parkinson's disease [5]. Nowadays we encounter in our practice Parkinson's disease patients suffering from hip joint osteoarthritis and avascular necrosis of femoral head, or complications of the hip arthroplasty implanted before the onset of Parkinson's disease such as symptomatic aseptic loosening or periprosthetic fracture of the femur [6]. Contemporary total hip arthroplasty is one of the highly efficient surgical techniques leading to improvement in the patient's quality of life [7]. Nonetheless, reports of the outcomes of elective total hip arthroplasty in patients with Parkinson's disease in the literature are sparse [8, 9]. But it is reasonable to be aware that orthopaedic surgeons will increasingly be required to evaluate the suitability of patients with Parkinson's disease for total hip arthroplasty.

The aim of the study was to compare the short- to mid-term clinical outcomes following elective and traumatic total hip arthroplasty in patients with Parkinson's disease focusing on the assessment of risks and benefits of surgery.

## 2. Materials and Methods

### 2.1. Patients

All patients with a confirmed diagnosis of Parkinson's disease having total hip arthroplasty at our institution between January 2005 and December 2012 were enrolled in a retrospective analysis. In total, 24 total hip arthroplasties were implanted in 23 patients, 8 men (35%) and 15 women (65%). The primary indication for surgery was osteoarthritis for 10 hips; these 10 patients comprise the “elective group” with a mean age at operation 74 years (65 to 82). Thirteen patients underwent total hip arthroplasty for proximal femoral fracture (one patient had hip fracture subsequently on both sides); they comprise the “hip fracture group” with a mean age at operation 76 years (67 to 83) (Table 1). The research was carried out in compliance with the Helsinki Declaration. Written informed consent was obtained from all patients included in the study.

### 2.2. Surgical Procedure

All the procedures were performed via a standard anterolateral Watson-Jones approach to the hip joint. In order to maximize sample size in the elective and hip fracture groups, implant design was not a controlled variable. The details of components with regard to stability of the implanted nonconstrained total hip arthroplasty are depicted in Table 2. All patients received prophylactic intravenous antibiotics for 24 hours postoperatively. Venous thromboprophylaxis was with low-molecular-weight heparin for 5 weeks. Standard postoperative rehabilitation as for any THA was started on the first postoperative day including mobilization in high vertical walker.

### 2.3. Outcome Assessments

Outcome measure analysed in the study included Charnley's modified Merle d'Aubigné and Postel scoring system [10]. As a result of Merle d'Aubigné and Postel scoring system is a composite score including objective clinical parameters different from pain, the pain score component of the Charnley's modified Merle d'Aubigné and Postel score was also evaluated separately. The disability caused by the Parkinson's disease was classified according to Hoehn and Yahr [11]. Furthermore, a functional status was based on assessment of independent ability to walk and was also analysed distinguishing between the following two findings [12]:

1. Maintained independent ability to walk: being able to walk without support from another person (with aids if necessary).
2. Not maintained: support from another person or use of a wheelchair required.

Complications were recorded throughout the follow-up period. All patients were followed up prospectively before surgery, at 6 months and 36 months postoperatively, and at the time of the latest follow-up.

In the elective group consisting of 10 hips, the mean follow-up was 82 (33–143) months. One patient in the elective group died at 33 months postoperatively from causes unrelated to the surgery (pulmonary tumor). For 14 hips in the hip fracture group, the mean follow-up was 54 (1–143) months. Five patients in the hip fracture group died during follow-up at 1, 3, 4, 11, and 30 months postoperatively.

### 2.4. Statistical Analysis

Measurement data were processed and statistically evaluated with the help of MS Excel 2013 (Microsoft Corp, Redmond WA, USA) and NCSS 2007 (Hintze, J. (2007). NCSS 2007. NCSS LLC, Kaysville, Utah, USA. https://www.ncss.com). Since the type of data of all the tested parameters is ordinal, we opted for using the Wilcoxon signed-rank test. A value of *P* < 0.05 was considered to be significant. For the purpose of comparison of the elective with the hip fracture group, only the data of the patients who survived until the last follow-up (median 82 months for the hip fracture and 72 months for the elective group) were used.

## 3. Results

**Figure 1:**
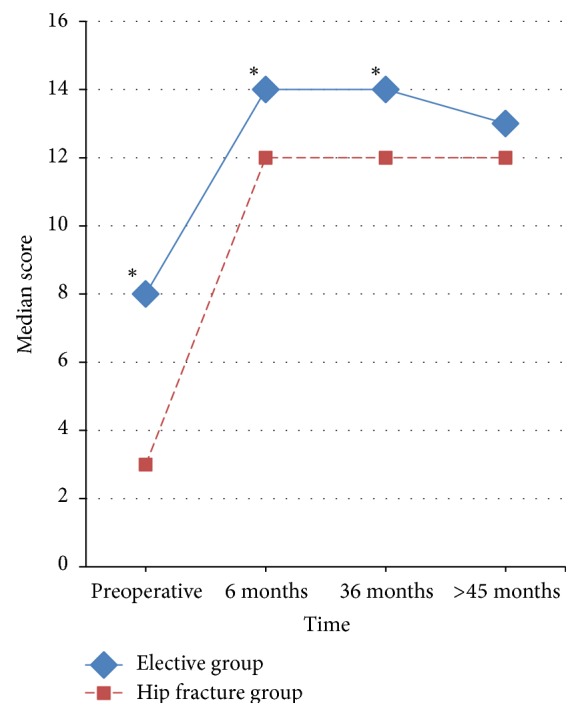
Line graph of Merle d'Aubigné and Postel scores by time in the elective and hip fracture group. Comparison of the progress of the medians of Merle d'Aubigné and Postel score during follow-up. The asterisk (**∗**) indicates the statistically significant difference between the compared groups.

The statistically significant difference between the medians of Merle d'Aubigné and Postel score in the elective and hip fracture group was recorded preoperatively, at 6 and 36 months after total hip arthroplasty. There was no statistically significant difference between the two groups during the last follow-up (Figure 1). The improvement in Merle d'Aubigné and Postel score preoperatively to 6 months postoperatively was statistically significant (*P* < 0.001) for both groups; there was no difference between the 6 months and 36 months and between the 36 months and latest follow-up Merle d'Aubigné and Postel scores in the elective group and in the hip fracture group too.

**Figure 2:**
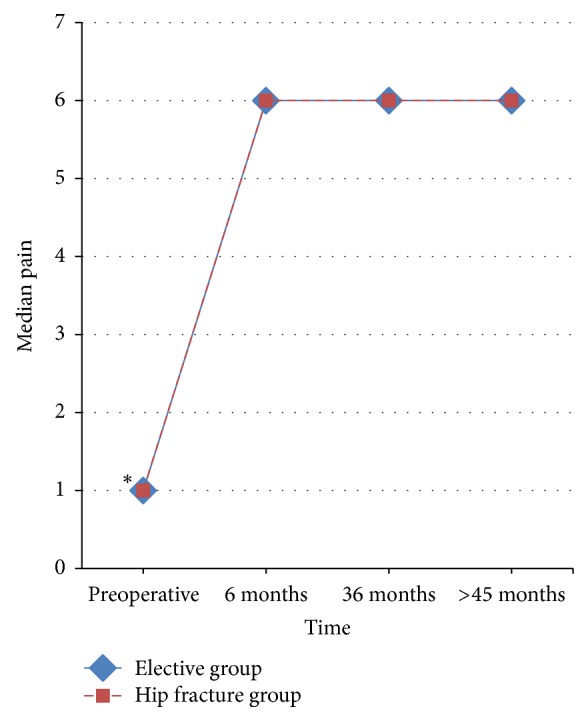
Line graph of pain scores by time in the elective and hip fracture group. Comparison of the progress of the medians of pain score during follow-up. The asterisk (**∗**) indicates the statistically significant difference between the compared groups.

The elective group had preoperatively significantly higher pain score when compared with the hip fracture group. There was no statistically significant difference between the two groups during the 6 months, 36 months, and the last follow-up (Figure 2). The improvement in pain score preoperatively to 6 months postoperatively was statistically significant (*P* < 0.001) for both groups with no differences between the 6 months and 36 months pain scores and between the 36 months and latest follow-up scores in the elective group and in the hip fracture group too. Of the 20 patients (21 hips) of both groups followed up at 6 months, 18 hips had no pain and 3 hips had only slight pain. Of the 17 patients (18 hips) of both groups followed up at 36 months, 17 hips had no pain and 1 hip had only slight pain. There was no pain observed in 18 hips of both groups at the time of latest follow-up.

Despite the improvement in Merle d'Aubigné and Postel and pain scores, disability related to Parkinson's disease increased during the follow-up; neurological progression was noted in 83% of all our patients. At the time of latest follow-up 57% of all the patients had progressed to functional stage IV or V (Table 3).

**Figure 3:**
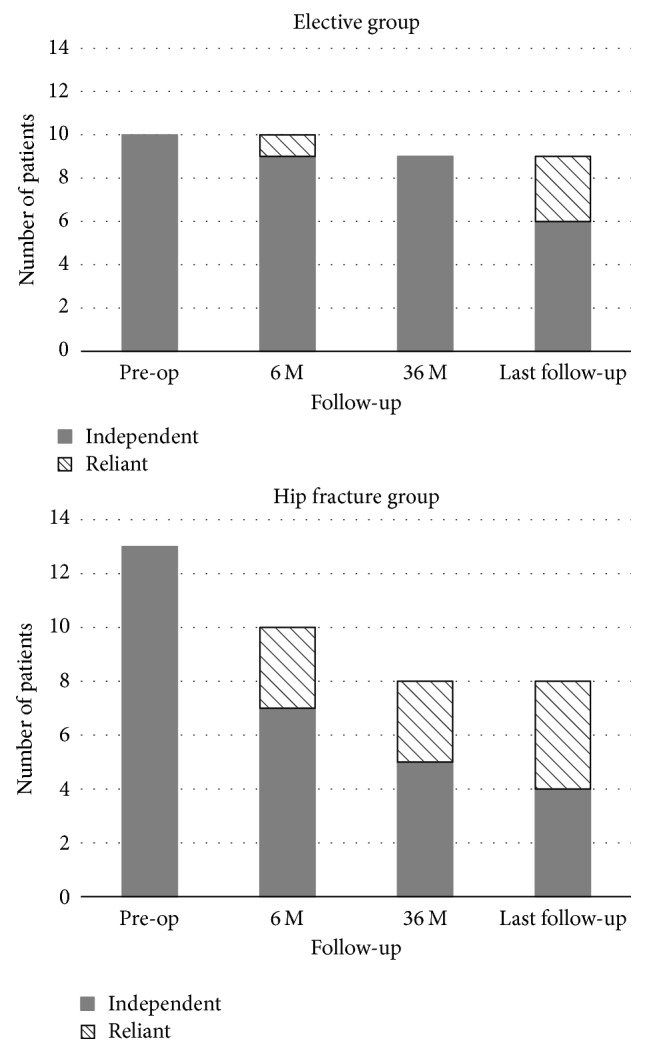
Distribution of independent and reliant patients in the elective and hip fracture groups during follow-up.

All patients in both groups were able to walk independently before surgery and functional status deteriorated over time as seen in Figure 3. Whereas 7 patients (54%) in the hip fracture group were able to walk independently, 9 patients (90%) in the elective group walked without support of another person at 6 months after surgery. Whereas 5 patients (38%) in the hip fracture group were able to walk without support of another person at 36 months after total hip arthroplasty, 9 patients (100%) in the elective group walked independently. At the time of the latest follow-up, 10 of living patients from both groups (43%) were still able to walk independently of another person with aid of forearm crutches.

The complications are listed in Table 4. Whereas the surgical complications were observed in both groups, the medical complications were seen mainly in patients undergoing total hip arthroplasty for femoral fracture. Two dislocations occurred within the first 3 months postoperatively, one dislocation in each group. Treatment was closed reduction in one patient of the elective group. One patient of the hip fracture group developed instability after a fatal cerebrovascular accident. Two periprosthetic fractures (one patient of hip fracture group fell 2 months after total hip arthroplasty and one patient of elective group 32 months after initial surgery) were treated operatively by osteosynthesis. One late periprosthetic infection occurred 26 months postoperatively in the hip fracture group and antimicrobial suppression was chosen because comorbidities did not allow additional surgery. Throughout the whole follow-up period, urinary tract infection and pneumonia were the most frequent medical complications in the hip fracture group (coincident respiratory and urinary tract infections were recorded in 4 patients) and were treated with antibiotics.

Four patients in the hip fracture group died during first year after surgery: three of pneumonia and one of cerebrovascular accident. The overall 1-year mortality for the hip fracture group was 28.6%.

## 4. Discussion

Total hip arthroplasty is one of the major successes of modern medicine [7]. As a result of progress in the implant designs and the precise surgical techniques, the indications for this procedure are continually expanding [13]. Total hip arthroplasty may be now considered also in patients with different neurological dysfunctions which would previously have been managed by salvage procedures. Several studies have evaluated the outcome of hemiarthroplasty in Parkinson's disease patients with hip fractures [14–16]. Unfortunately these surveys are not accurately applicable to an elective total hip arthroplasty. In order to support the current evidence regarding the outcome of total hip arthroplasty in Parkinson's disease patients we carried out a present study.

Our study demonstrates a clear improvement in hip pain following total hip arthroplasty in patients with Parkinson's disease as supported by improved Merle d'Aubigné and Postel and pain scores. This improvement was maintained from 6 months after surgery to the latest follow-up in the elective and hip fracture group. Although the functional status of Parkinson's disease patients deteriorated over time, we observed that patients in the elective group benefited from excellent pain relief and were able to walk without support of another person at 36 months after total hip arthroplasty which was indicated for osteoarthritis with severe hip joint pain preventing activities of daily living. Ten of living patients from both groups (43%) were still able to walk without support of another person at the time of the latest follow-up.

Although variable mortality following hip fracture in Parkinson's disease patients has been reported, we found it comparable with 1-year mortality published in non-Parkinson's disease patients [3, 17]. However, the morbidity with an increased complication rate was observed in our hip fracture group [18]. The most frequently recorded medical complication (64% of patients in the hip fracture group throughout the whole follow-up period) was urinary tract infection. The neurogenic detrusor overactivity is common in Parkinson's disease patients (45–93%) and bladder dysfunction plays important role in development of urinary tract infection [19]. In large retrospective study of neurogenic bladder patients, more than one-third (36%) of patients were diagnosed with a lower urinary tract infection at least one year after neurogenic bladder diagnosis [20].

Obstructive respiratory pattern due to neuromuscular dysfunction predisposes to retained secretion, atelectasis, and pulmonary infection [21]. We found pneumonia in 29% of Parkinson's disease patients with femoral fractures. High pulmonary infection rates (40–43%) were reported also by Eventov et al. and Staeheli et al. [15, 16]. Immobility, constant pressure, friction, reduced muscle mass, and low skin turgor contribute to development of pressure sores. Historically, high rates of pressure sores (25–49%) were published in Parkinson's disease patients after hip fracture [14, 15]. Despite early mobilization, we observed pressure sores in 12.5% of Parkinson's disease patients after total hip arthroplasty.

The musculoskeletal manifestation of Parkinson's disease include tremor, rigidity, contractures, bradykinesia, dystonia, and postural instability which theoretically predispose patients to dislocation of the hip [13]. Historically, high rate of hemiarthroplasty dislocation (37%) was published in Parkinson's disease patients who were not mobilized in the first week after hip fracture surgery [14]. The results in these patients after surgery for hip fractures were poor, mainly because of medical complications with high rates of morbidity and mortality. Later studies described lower rates of hemiarthroplasty dislocation in Parkinson's disease patients (2–11%) suggesting that these better outcomes may have been due to pharmacotherapy aimed at maintaining muscle tone [16, 22].

Recently Parkinson's disease patients had an approximately twofold risk of hip dislocation [23]. Adduction contracture is an important finding in these patients which can be overlooked during the procedure [6]. Due to pain and muscle spasm following hip fracture, it is not possible to assess the hip abduction prior to surgery. For this reason, careful intraoperative hip stability testing consisting of extension with external rotation motions, subsequent flexion with internal rotation motions, and check for telescoping of the components should be emphasized [24]. To prevent instability, adductor or psoas tenotomies for severe flexion contracture were recommended [8]. Based on thorough intraoperative assessment, the stability of all our total hip arthroplasties was adequate and no pelvifemoral muscle contractures were observed. Therefore adductor tenotomies were thought to be not necessary to prevent hip joint instability in our patients.

Despite early mobilization started on the first postoperative day, intensive postoperative rehabilitation, and patient's care, the dislocation rate of nonconstrained total hip arthroplasty in our study (8.3%) is comparable with recently reported rate in nationwide registry-based case-controlled study (6.1%) [23]. Both are significantly greater than has been published in patients without neurological dysfunction [25]. Therefore certain innovative implant designs, such as dual mobility acetabular cup and large-diameter heads, should be considered to reduce the incidence of dislocation in patients with Parkinson's disease [26, 27].

Most Parkinson's disease patients experience falls as a result of disease symptoms and many have recurrent episodes [28]. It was estimated that 60.5% of patients with Parkinson disease experience at least one episode and 39% have recurrent falls [29]. The high frequency of falls consequently contributes to the increased fracture risk. Falls in the period after total hip arthroplasty may result in serious periprosthetic fractures of the femur as is seen in 8.3% of hip joints in our study. Surgical treatment of periprosthetic fractures belongs to the most difficult orthopaedic procedures due to the extensive surgery with increased blood loss, high frequency of other complications, and series of unfavorable outcomes, such as disability and death [30].

With increasing prevalence of Parkinson's disease, falls and fractures are anticipated to have a major impact on health care systems in the coming decades [28]. Severity of disease and functional impairment might be substantial determinants of the risk of falls and fractures. However, neurodegenerative diseases and especially cognitive disorders substantially compromise individuals' physical reserves important for adaptation to changes in health state and acute stress, such as hip fracture [23]. In addition, patients with Parkinson's disease have poorer initial physical condition. In the setting of acute trauma for displaced femoral neck fractures, our study did not entirely support total hip arthroplasty in Parkinson's disease patients. During the period of the study, hemiarthroplasty was routinely used in moderate to low functioning elderly patients with displaced femoral neck fractures. Based on the results of our study, Parkinson's disease patients with hip fractures have poorer prognosis due to the disease progression with inevitable functional disability and therefore hemiarthroplasty remains an appropriate option [31].

This retrospective study is limited by the small patient numbers and short duration of follow-up. Nevertheless the follow-up was sufficient to reveal a clinical outcome following elective and traumatic total hip arthroplasty in patients with Parkinson's disease. Total hip arthroplasty is a viable alternative for these patients if the surgery is individualized and carefully planned. A multidisciplinary team comprised of health professionals, including the neurologist, geriatrician, and physiotherapist, should be involved to maximize patient outcome [32]. Early discussion with the patient and his family regarding the difficulty of prolonged rehabilitation process, as well as the potential need of prolonged stay in nursing home, should be emphasized in effort to optimize surgical outcomes in Parkinson's disease patients [5]. Before an elective procedure, tremor and other symptoms related to Parkinson's disease should be well controlled to minimize postoperative complications and enhance rehabilitation process. To be recognized as a potential key element for arthroplasty success, rehabilitation process should be started preoperatively with subsequent early postoperative mobilization and physical therapy [33]. The patients should be also carefully monitored for common complications, such as urinary tract infection, pulmonary infection, and pressure sores [5]. Finally, improving the patient bone density or preventing bone loss is important before joint arthroplasty is considered in Parkinson's disease patients.

## 5. Conclusions

In conclusion, total hip arthroplasty in patients with Parkinson's disease is challenging due to higher risk of medical complications which were seen mainly in patients with hip fracture. Excellent pain relief with preserved walking ability without support of another person and acceptable complication profile was observed in Parkinson's disease patients at 36 months after elective total hip arthroplasty. Elective total hip arthroplasty may be indicated in patients with Parkinson's disease after careful and individualized planning.

## Figures and Tables

**Table 1:** Demographics of patients included in the study.

	Elective group	Hip fracture group
Number of patients	10	13
Number of hips	10	14
Mean age in years (range)	74 (65–82)	76 (67–83)
Gender		
Female	8	7
Male	2	6
Right side	5	5
Left side	5	9
ASA score, average	2.50	2.62
Hoehn-Yahr scale, average	2.30	2.31

**Table 2:** Details of implants used in the study.

	Elective group	Hip fracture group
Acetabular component		
Cemented	8	13
Cementless	2	1
Acetabular liner		
Standard	7	8
With elevated rim/lipped	3	6
Femoral stem		
Cemented	9	12
Cementless	1	2
Head diameter		
28 mm	7	13
32 mm	3	1

**Table:** (a) Elective group

Case	A	B	C	D	E	F	G	H	I	J
1	2	75	2	33	3/4	1/2	1 + 4	70/80	30/20	7 + 4
2	2	71	2	123	2/5	1/2	1 + 5	70/100	0/30	6 + 8
3	1	66	3	143	2/3	1/1	2 + 4	95/90	35/40	10 + 3
4	1	77	3	99	2/2	1/1	1 + 5	90/95	20/20	8 + 6
5	2	70	3	132	2/4	1/2	1 + 5	70/90	30/30	8 + 7
6	2	82	2	60	2/2	1/1	1 + 5	60/90	15/30	6 + 8
7	2	77	3	72	3/3	1/1	2 + 4	120/90	30/40	9 + 4
8	2	82	2	52	2/3	1/1	2 + 3	95/90	30/30	12 + 0
9	2	65	2	53	3/4	1/1	1 + 5	80/90	25/30	7 + 6
10	2	74	3	48	2/3	1/2	2 + 4	85/120	20/30	10 + 4

**Table:** (b) Hip fracture group

Case	A	B	C	D	E	F	G	H	I	J
1	1	70	3	1	3/5	1/2	1 + *∗*	*∗*/*∗∗*	*∗*/*∗∗*	3 + *∗*
2	1	75	3	4	3/5	1/2	1 + *∗*	*∗*/*∗∗*	*∗*/*∗∗*	3 + *∗*
3	2	80	3	143	2/3	1/1	1 + 5	*∗*/95	*∗*/30	3 + 12
4	2	73	2	93	2/5	1/2	1 + 5	*∗*/90	*∗*/30	3 + 9
5 left	2	80	3	53	3/3	1/1	1 + 5	*∗*/80	*∗*/20	3 + 9
5 right	2	80	3	53	3/3	1/1	1 + 4	*∗*/70	*∗*/20	3 + 8
6	2	78	2	85	2/4	1/2	1 + 5	*∗*/90	*∗*/30	3 + 10
7	2	67	3	82	2/5	1/2	1 + 5	*∗*/90	*∗*/15	3 + 9
8	1	83	3	87	3/5	1/2	1 + 5	*∗*/90	*∗*/40	3 + 10
9	1	77	3	11	2/5	1/2	1 + 5	*∗*/90	*∗*/15	3 + 9
10	2	75	2	3	2/5	1/2	1 + *∗*	*∗*/*∗∗*	*∗*/*∗∗*	3 + *∗*
11	1	75	2	61	2/3	1/1	1 + 5	*∗*/70	*∗*/25	3 + 9
12	2	74	2	45	1/2	1/1	1 + 5	*∗*/60	*∗*/30	3 + 9
13	1	82	3	30	3/4	1/2	1 + 5	*∗*/60	*∗*/25	3 + 9

A: gender: 1: male; 2: female; B: age at total hip arthroplasty (years); C: ASA score; D: follow-up (months); E: Hoehn and Yahr functional stage preoperatively/at the time of latest follow-up; F: functional status preoperatively/at the time of latest follow-up: 1: maintained independent ability to walk; 2: not maintained independent ability to walk; G: preoperative pain score ± the postoperative increase at 6 months postoperatively (points); *∗* the data were not available (short follow-up); H: hip flexion preoperatively/at 6 months postoperatively (degrees); *∗* indicates that the hip joint was not moved in sagittal plane due to fracture; *∗∗* indicates that the data were not available (short follow-up); I: hip abduction preoperatively/at 6 months postoperatively (degrees); *∗* indicates that the hip joint was not moved in frontal plane due to fracture; *∗∗* indicates that the data were not available (short follow-up); J: preoperative Merle d'Aubigné and Postel score ± the postoperative increase at 6 months postoperatively (points); *∗* indicates that the data were not available (short follow-up).

**Table 4:** Complications.

Complication	Elective group (10 hips; 10 patients)	Hip fracture group (14 hips; 13 patients)
Dislocation	1	1
Periprosthetic fracture	1	1
Periprosthetic infection	0	1
Cerebrovascular accident	0	1
Postoperative confusion	0	2
Urinary tract infection	0	9
Pneumonia	0	4
Vulvovaginitis	1	0
Pressure sores	1	2
Decompensation of diabetes mellitus	1	0

*Total*	*5*	*21*

Six patients (five in the hip fracture group) had two complications each. Two patients in the hip fracture group had four complications.
